# Comparative Study of the Effects of Citral on the Growth and Injury of *Listeria innocua* and *Listeria monocytogenes* Cells

**DOI:** 10.1371/journal.pone.0114026

**Published:** 2015-02-02

**Authors:** Angela B. Silva-Angulo, Surama F. Zanini, Amauri Rosenthal, Dolores Rodrigo, Günter Klein, Antonio Martínez

**Affiliations:** 1 Departamento de Biotecnologia Agrolimentaria, Biopolis S.L., Avda. Agustín Escardino, 9, 46980 Paterna, Spain; 2 Espirito Santo Federal University, Department of Veterinary Medicine, Alegre, Brazil; 3 Embrapa Food Technology, Av. das Américas 23020–470, Rio de Janeiro, Rio de Janeiro, Brazil; 4 Department of Preservation and Food Quality, Instituto de Agroquímica y Tecnología de Alimentos (IATA-CSIC), Valencia, Spain; 5 Institute of Food Quality and Food Safety, University of Veterinary Medicine, Hannover, Germany; Indian Institute of Science, INDIA

## Abstract

This study investigates the effect of citral on growth and on the occurrence of sublethal damage in *Listeria innocua* Serovar 6a (CECT 910) and *Listeria monocytogenes* Serovar 4b (CECT 4032) cells that were exposed to citral as a natural antimicrobial agent. Two initial inoculum concentrations were considered in this investigation: 10^2^ and 10^6^ cfu/mL. Citral exhibited antilisterial activity against *L. innocua* and *L. monocytogenes*, and the observed effects were dependent on the concentration of citral present in the culture medium (0, 0.150 and 0.250 μL/mL) (p ≤ 0.05). *L. innocua* had a shorter lag phase than *L. monocytogenes*, and the two species had nearly identical maximum specific growth rates. These results indicate that *L. innocua* could be used as surrogate for *L. monocytogenes* when testing the effects of this antimicrobial. Significant differences in the lag phase and growth rate were observed between the small and large inoculum concentration (p ≤ 0.05). Citral-treated *L. innocua* and *L. monocytogenes* that were recovered on selective medium (i.e., TSA-YE-SC) had a shorter lag phase and a higher maximum specific growth rate than cells that were recovered on non-selective medium (i.e., TSA-YE) (p ≤ 0.05). This result suggests that damage occurs at sublethal concentrations of citral.

## Introduction

*Listeria monocytogenes* can grow under conditions (low temperature) that prevent the survival of other foodborne pathogenic bacteria. Therefore, this bacterium is an important target organism for elimination from the food supply. As laid down in Commission Regulation (EC) No 2073/2005, *L. monocytogenes* levels should be lower than 100 colony forming units per gram throughout the shelf life of a food product if the product does not allow growth or if the product is not intended for an at-risk population [1]

*L. innocua* is the most commonly detected species of *Listeria* in the food industry and it is not a pathogenic form of *Listeria*. The food contamination by other species of *Listeria* indicated that these non pathogenic *Listeria* possess characteristics that enable their development, since all *Listeria* species have high homology between the genomes (which makes them very similar phenotypically), besides having the same ecology [2]

Although *L. innocua* has been proposed as a surrogate for *Listeria monocytogenes* [3], results obtained from models that used surrogates for *L. monocytogenes* strains in cases where preservation is achieved using a natural antimicrobial compound, could not be extended to *L. monocytogenes*. Therefore, the suitability of *L. innocua* as a surrogate must be verified to ensure efficient elimination of this dangerous pathogen.

Mild food processing methods must efficiently suppress the growth and/or reduce the number of pathogenic bacteria, such as *L. monocytogenes*. Biopreservatives, such as essential oils, are nonthermal preservation technologies that can be used alone or in combination with other nonthermal technologies, such as pulsed electric fields, high pressure or ultraviolet radiation [4], [5].

Citral is a monoterpenoid aldehyde [6] and often it is present in the form of the stereoisomers neral and geranial [7]. It has been shown to be present in the leaves and fruits of several plant species including myrtle trees, basil, lemon, lime, lemongrass, orange and bergamot [8], [6]. The precise targets of terpenoids are not yet completely understood although antimicrobial effects of citrus oils have been shown to be bacteriostatic [9]. It is known that there is damage to the cell by increasing the cell membrane permeability, changing cell morphology and decreasing ATP synthesis because the membrane potential is the driving force of ATP synthesis; thus, the reduction of internal ATP is coupled with the loss of membrane potential. Also, in the presence of the citrus essential oil blend, this delicate balance is lost and due to the disruption of the membrane integrity, there is a loss of control of the H^+^ ion gradients [10].

These preservation treatments could also produce a large number of damaged cells when administered at concentrations below the MIC (minimum inhibitory concentration). Because damaged cells can repair their injuries during the storage period those cells should be considered in the design of preservation processes. In addition, these cells could acquire new abilities during the repair process, such as resistance to antimicrobials, antibiotics or other preservation technologies [11], [12], [13]. Consequently, sub-lethal injury is an important factor that should be considered in evaluating the efficacy of any food preservation method. Thus, evaluating the behaviour of *L. innocua* as a surrogate for *L. monocytogenes* is an important task.

The majority of previous growth studies use a fixed inoculum level without considering the effects of variations in inoculum concentration on growth characteristics. In the presence of specific stresses and under suboptimal conditions, inoculum size affects the duration of the lag [14], [15]. This observation is important because common predictive microbial growth models are done with initial concentrations of bacteria higher than 10^3^ cfu/mL.

The aim of this study was to evaluate the effect of citral on growth kinetics and its contribution producing damaged cells in *L. monocytogenes* Serovar 4b (CECT 4032) and *L. innocua* Serovar 6a (CECT 910) at two initial inoculum concentrations: 10^2^ and 10^6^ cfu/mL. The behaviour of *Listeria innocua* and *Listeria monocytogenes* was compared to evaluate the use of *Listeria innocua* as a surrogate for *Listeria monocytogenes* when this antimicrobial substance is used to control microbial growth at levels below the minimal inhibitory concentration (MIC).

## Materials and Methods

### Chemicals

The reagent 95% citral (3,7-dimethyl-2,6-octadienal), which contains a mixture of cis and trans isomers, was purchased from Sigma Aldrich Company Ltd, Steinheim, Westphalia, Germany.

### Bacterial strains and growth conditions

Foodborne *L. monocytogenes* Serovar 4b (CECT 4032) isolated from soft cheese and *L. innocua* Serovar 6a (CECT 910) were obtained from a pure lyophilized culture supplied by the Spanish Type Culture Collection. Glycerinated stock vials of *L. monocytogenes* and *L. innocua* were generated following the method described by [16]. During this investigation, stock cultures were maintained in cryovials at a concentration of approximately 8.5 × 10^8^ colony forming units/mL (cfu/mL) and a temperature of -80°C. For both species, the average cell density of the vials was established by viable plate counting, using buffered peptone water (Scharlau Chemie S. A., Barcelona, Spain) to dilute the samples.

Bacterial broth subcultures were prepared from stock cultures by inoculating 200 μL of *Listeria monocytogenes* Serovar 4b (CECT 4032) or *Listeria innocua* Serovar 6a (CECT 910) into a sterile flask containing 6 mL of Tryptone Soya Broth (TSB) (Scharlab Chemie S.A., Barcelona, Spain) and incubating the flasks at 37°C for 12 h to obtain an 8 log_10_ cfu/mL suspension. The cell concentration was verified by viable plate count.

### Determination of antimicrobial activity

Bacteria were grown in TSB supplemented with different concentrations of citral. To see the occurrence of damaged cells, low doses of citral were used according to previous studies of growth kinetics carried out by [17], [18], [19]. Injured cells can be sensitive to this agent but may still have the potencial to repair. This natural antimicrobial agent was tested in a broth growth medium at the following levels: 0.0 μL/mL of citral (i.e., control), 0.150 μL/mL of citral and 0.250 μL/mL of citral. Briefly, the compound to be tested was added at the indicated concentrations to 20 mL of TSB in a sterile flask. An aliquot of an overnight culture of *L. monocytogenes* or *L. innocua* was added to each sample to obtain approximately 10^2^ and 10^6^ colony-forming units (cfu) per mL. Each culture was incubated under agitation at 37°C for 30 h.

**Counts of viable cells**. After treatment, *L. innocua* and *L. monocytogenes* growth was estimated by plate count on Tryptone Soya Agar (TSA) (Scharlab Chemie S.A., Barcelona, Spain) supplemented with 0.6% yeast extract (TSA-YE). Growth curves were obtained by viable plate count, with concentrations of 10^2^ and 10^6^ cfu/mL at time zero. To obtain growth curves, samples of the culture were diluted in buffered peptone water (Scharlau Chemie S.A., Barcelona, Spain) and pour-plated onto Tryptic Soy Agar (TSA) (Scharlab Chemie S.A., Barcelona, Spain) at 0, 1, 2, 4, 6, 8, 10, 12, 15, 20, 25 and 30 hours. At least three separate replicates were performed for each tested condition.

The plates were incubated at 37°C for 48 h, and the number of colony forming units was subsequently determined by plate count.

### Bacterial growth models and calculation of kinetic parameters

To determine the kinetics of microbial growth, non-linear regression was used to fit the experimental data to the Gompertz equation, as described by [20]. The mathematic expression of this equation is as follows:

$$L_{10}\left(N_{t}\right)=A+Cexp\left\{-exp^{\left[-B\left(t-M\right)\right]}\right\}$$

where N_t_ represents the number of microorganisms at time t (cfu/mL); A represents the inferior asymptote value (log_10_ (cfu/mL)); C represents the difference between the curve asymptotes (log_10_ (cfu/mL); B represents the relative growth rate when t = M ((log_10_ (cfu/mL))/h); M represents the elapsed time until the maximum growth rate is reached (h); and e represents the number e.

This model was selected based on previous studies in which intervention using natural antimicrobial compounds was modelled [5], [21], [22].

A, B, C and M were used to calculate the kinetic parameters lag time (λ; h) and maximum growth rate (μ_max_) ((log_10_ (cfu/mL))/h), using the equations described by [20], [23].

The goodness of fit was measured based on the mean square error (MSE) and the corrected determination coefficient (corrected R^2^) for each set of data.

### Determination of the percentage of injured cells

To estimate the number of sublethally injured cells, a separate set of experiments was performed. At specific time intervals, 0.1 mL of *L. monocytogenes* or *L. innocua* samples that were adequately diluted in sterile 0.1% (w/v) peptone water (Scharlau Chemie S. A., Barcelona, Spain) were pour-plated onto TSA-YE supplemented with 5% (w/v) NaCl. The non-selective TSA-YE medium was expected to support the growth of both uninjured and antimicrobial-injured cells, whereas the selective TSA-YE medium supplemented with 5% (w/v) of sodium chloride (TSA-YE-SC) agar was expected to support the growth of uninjured populations [24]. Thus, the percentage of sublethal damage produced after treatment with each antimicrobial could be determined. The loss of tolerance to the presence of sodium chloride and the resulting inability to grow on selective media are attributed to damage that affects the function and/or the integrity of the cytoplasmic membrane [25].

The samples that were recovered in selective and nonselective media were incubated for 48 h at 37°C. The number of colony forming units was then determined by plate count.

The percentage of sublethally injured cells was estimated using the following equation [26], [27]:

$$\left[1-\left(\frac{countonTSA\_YE\_SC}{countonTSA\_YE}\right)\right]*100$$

where samples were pour-plated onto nonselective medium (TSA-YE) and selective medium (TSA-YE-SC). Therefore, the proportion of sublethally injured cells was estimated by comparing the number of log_10_ cycles of inactivation obtained after plating the antimicrobial agent-treated cells onto the nonselective and selective media [24].

The error bars in the figures indicate the mean ± standard deviations of the data obtained from at least three repetitions.

### Statistical Analysis of Data

Statistical analysis was performed using Statgraphics Centurion XV software (StatPoint Technologies, Inc., Warrenton, VA, USA). This analysis included an ANOVA to detect significant differences in growth kinetics parameters and in the percentage of damage to *L. monocytogenes* and *L. innocua* cells after exposure to citral concentrations (p ≤ 0.05). When necessary, a multiple range test was also applied to identify the levels of each factor that were perceptibly different (p ≤ 0.05). Fisher’s LSD (Least Significant Difference) test was used to compare the mean values of the data (p ≤ 0.05). At least three repetitions were performed for each treatment. Colony counts were converted into logarithm values to identify differences that were significant at the 95% (p *≤* 0.05) confidence limit.

## Results and Discussion

Citrus oils not only lend themselves to use in food but also are generally recognised as safe (GRAS) and have been found to be inhibitory both in direct oil and vapour form against a range of both Gram-positive and Gram-negative bacteria ([8]. Lemon, sweet orange and bergamot and their components, linalool and citral were found to have antimicrobial effects both in direct oil and vapour form against *Campylobacter jejuni*, *E. coli*, *E. coli O157*, *L. monocytogenes*, *Bacillus cereus*, *S. aureus* [8], [28], *Enterococcus faecalis* and *Enterococcus faecium* by increase of cell permeability [9], [10]. Other report showed inhibition against *E. coli* and *S.* Typhimurium from lemon oil and citral [29].

**Figure 1:**
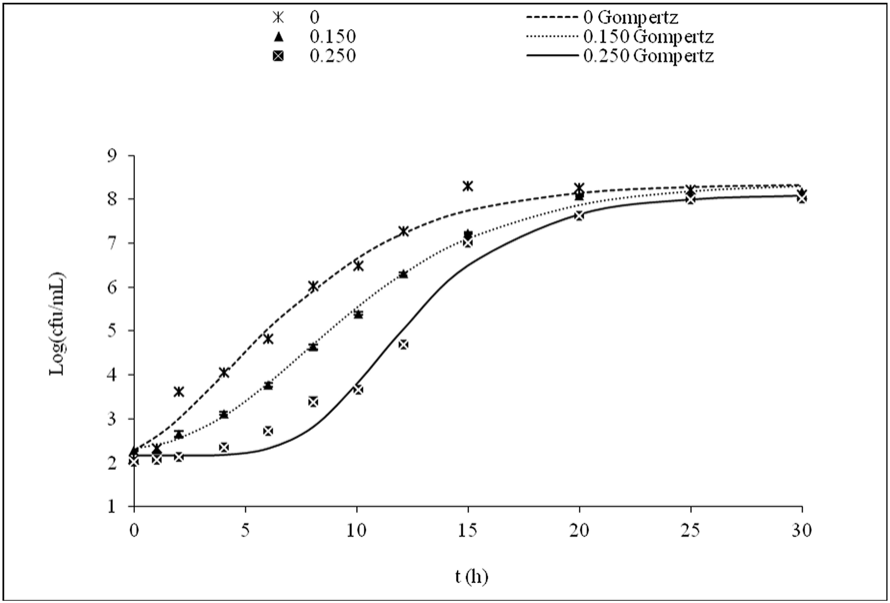
*L. innocua* growth curves in reference medium in the presence of different concentrations of citral (μL/mL) with N_0_ = 10^2^ Log(cfu/mL). The lines represent the fit of the experimental data to the modified Gompertz model. The standard deviation associated with each average value is expressed by error bars.

**Figure 2:**
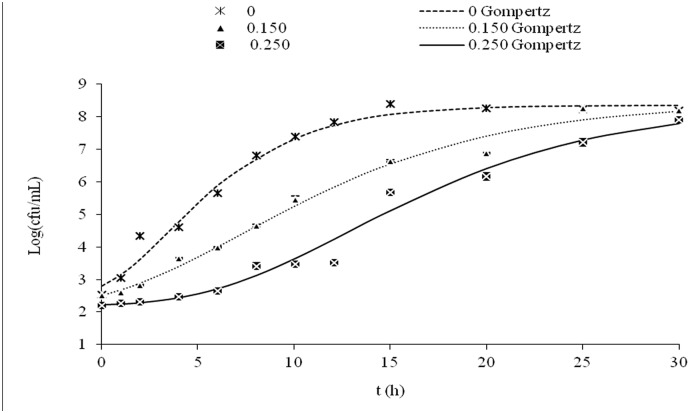
. *L. monocytogenes* growth curves in reference medium in the presence of different concentrations of citral (μL/mL) with N_0_ = 10^2^ Log(cfu/mL). The lines represent the fit of the experimental data to the modified Gompertz model. The standard deviation associated with each average value is expressed by error bars.

Experimental *L. innocua* and *L. monocytogenes* growth curves were obtained using inoculum concentrations of 10^2^ and 10^6^ cfu/mL in TSB alone or in TSB supplemented with different concentrations of citral that were below the MIC. Growth curves were fitted to the Gompertz model, and the lag phase and the specific growth rate were calculated (Tables 1 and 2). Figs. 1 and 2 show examples of fitted growth curves. The statistical analysis revealed adjusted correlation coefficients (R^2^) for *L. innocua* and *L. monocytogenes* growth that were greater than 0.97 and 0.93, respectively, indicating that the Gompertz model was a quite good model for this study.

**Table 1:** Mean values ± standard error of the duration of the lag phase (λ) and of the maximum specific growth rate (μ_max_) of *Listeria innocua* cells that were recovered on TSA-YE medium as a function of the initial inoculum size (i.e., 10^2^ cfu/mL or 10^6^ cfu/mL) and the concentration of citral (μL/mL).

**Citral (μL/mL)**	**λ ± SE (h)**		**μ_max_ ± SE (log_10_ (cfu/mL))/h**	
	**10^2^ cfu/mL**	**10^6^ cfu/mL**	**10^2^ cfu/mL**	**10^6^ cfu/mL**
**0.0**	0.305 ± 0.03^A^ ^a^	0.287 ± 0.07^A^ ^a^	0.547 ± 0.02^A^ ^a^	0.529 ± 0.04^A^ ^a^
**0.150**	2.180 ± 0.01^B^ ^b^	1.832 ± 0.01^B^ ^a^	0.440 ± 0.01^B^ ^a^	0.432 ± 0.01^B^ ^a^
**0.250**	6.012 ± 0.01^C^ ^b^	4.100 ± 0.03^C^ ^a^	0.202 ± 0.04^C^ ^b^	0.101 ± 0.03^C^ ^a^

^A–C^Mean values followed by different letters in the same column differ significantly by Fisher’s LSD test (p ≤ 0.05).

^a, b^Mean values followed by different letters in the same row differ significantly by Fisher’s LSD test (p ≤ 0.05).

**Table 2:** Mean values ± standard error of the duration of the lag phase (λ) and the maximum specific growth rate (μ_max_) of *Listeria monocytogenes* cells that were recovered on TSA-YE medium as a function of the initial inoculum size (i.e., 10^2^ cfu/mL or 10^6^ cfu/mL) and the concentration of citral (μL/mL).

**Citral (μL/mL)**	**λ ± SE (h)**		**μ_max_ ± SE (log_10_ (cfu/mL))/h**	
	**10^2^ cfu/mL**	**10^6^ cfu/mL**	**10^2^ cfu/mL**	**10^6^ cfu/mL**
**0.0**	0.533± 0.03^A^ ^a^	0.500 ± 0.04^A^ ^a^	0.590 ± 0.04^A^ ^a^	0.571 ± 0.03^A^ ^a^
**0.150**	2.569 ± 0.04^B^ ^b^	1.259 ± 0.06^B^ ^a^	0.470 ± 0.01^B^ ^a^	0.448 ± 0.01^B^ ^a^
**0.250**	8.094 ± 0.04^C^ ^b^	4.754 ± 0.05^C^ ^a^	0.181 ± 0.01^C^ ^b^	0.102 ± 0.02^C^ ^a^

^A–C^Mean values followed by different letters in the same column differ significantly by Fisher’s LSD test (p ≤ 0.05).

^a, b^Mean values followed by different letters in the same row differ significantly by Fisher’s LSD test (p ≤ 0.05).

The kinetic behaviour of *L. innocua* and *L. monocytogenes* was characterised based on the time needed to adapt to the environment (i.e., the lag phase (λ)) and on the maximum specific growth rate (μ_max_). In our study, the mean values of the lag phase and the maximum specific growth rate (Tables 1 and 2) are consistent with the distributions of the 1,865 previously reported μ_max_ values and the 1,294 previously reported λ values that were considered by Augustin and [30] in their study of growth parameters for *Listeria monocytogenes*.

A comparison of the parameters obtained in this study demonstrated that citral exhibited activity against *L. innocua* and *L. monocytogenes* and that the observed effects were dependent on the concentration of citral present in the culture medium (p ≤ 0.05). Higher concentrations of citral resulted in a longer lag phase and a lower specific growth rate (Tables 1 and 2). Therefore, the observed bacteriostatic effect was understood as an increased lag phase accompanied by a reduced specific growth rate. This behaviour was also described by [31].

A comparison of the behaviour of these microorganisms after citral exposure indicated that the lag phase of *Listeria innocua* was shorter than that of *Listeria monocytogenes* at all concentrations tested. However, the maximum specific growth rate was similar for both microorganisms. The difference within the lag phase duration between *Listeria* species indicates that *L. monocytogenes* was more sensitive to citral exposure than *L. innocua* because *L. innocua* presents a shorter period of latency. These results suggest that the use of *Listeria innocua* as a surrogate for *Listeria monocytogenes* when testing the use of citral as an antimicrobial substance in food products is appropriate because *Listeria innocua* represents a worse scenario providing a safety margin due to its shorter lag phase. Previous studies reported that the presence of *L. innocua* is an indicative of greater likelihood of *L. monocytogenes* ocurrence [2], [32].

Commission Regulation (EC) No 2073/2005 on microbiological criteria for foodstuffs, applicable from 1 January 2006, lays down food safety criteria for certain important foodborne bacteria, their toxins and metabolites, such as *Listeria monocytogenes*. These criteria are applicable to products placed on the market during their entire shelf-life. The Scientific Committee on Veterinary Measures relating to Public Health (SCVPH) recommended that it is an objective to keep the concentration of *Listeria monocytogenes* in food below of 100 cfu/g during products shelf-life on market. Due to manipulation and coolant failures their multiplication is possible [33]. Most cases of listeriosis are associated with the consumption of foods that are above the allowed limit [34].

The minimum infective dose of *Listeria monocytogenes* in humans is not known but appears to be of the order of 10^3^ CFU [35]. Data collected in outbreaks of listeriosis suggest that incriminated foods contained high counts of *Listeria monocytogenes* about 10^6^ [36] which emphasizes the need to minimize human exposure to high populations of bacteria. Therefore, it is appropriate to evaluate the effect of citral in a dose reported in outbreaks of listeriosis.

To evaluate the influence of the inoculum size on the activity of citral, the experiment was conducted using two initial population concentrations: 10^2^ cfu/mL, which is the maximum number of organisms allowed in a food product according to EU Regulations, and 10^6^ cfu/mL which is a quite frequent in outbreaks of listeriosis. Studies performed using an initial inoculum size of 10^2^ cfu/mL indicated that the use of 0.150 μL/mL and 0.250 μL/mL of citral increased λ by 1.65 h and 5.36 h, respectively, for *Listeria innocua* and 1.69 h and 7.02 h, respectively, for *L. monocytogenes* and decreased μ_max_ by 0.076 log/h and 0.363 log/h, respectively, for *Listeria innocua* and 0.098 log/h and 0.397 log/h, respectively, for *L. monocytogenes*, as presented in Tables 1 and 2. At an initial inoculum concentration of 10^6^ cfu/mL, the use of 0.150 μL/mL and 0.250 μL/mL of citral increased λ by 1.06 h and 3.71 h, respectively, for *Listeria innocua* and 0.61 h and 3.87 h, respectively, for *L. monocytogenes* and decreased μ_max_ by 0.071 log/h and 0.419 log/h, respectively, for *Listeria innocua* and 0.103 log/h and 0.454 log/h, respectively, for *L. monocytogenes*, as presented in Tables 1 and 2.

These results indicate that the inoculum concentration affected the lag phase, with a longer lag phase observed using a lower inoculum size (p≤0.05). In contrast, the maximum specific growth rate appears to be unaffected by the inoculum size, regardless of the microorganism considered (p>0.05) except for the higher citral concentration studied. This result appears to indicate that under unfavourable growth conditions, the inoculum size influences the growth kinetics. The inoculum size effect, which is observed only with severely stressed cells, could be explained by an increase in the variation of the lag times of individual cells when the cells are stressed [37], [38]. ]. It has been reported that the lag time of *Listeria monocytogenes* growing under suboptimal conditions was extended when the inoculum was severely stressed by starvation and when the inoculum size was very small [30]. A similar result was obtained by [15]. Although the maximum specific growth rate is generally assumed to be independent of the inoculum size, this parameter could be a function of the population density under unfavourable conditions [39].

Recent studies conducted in *E. coli* by [40] demonstrated that a larger initial inoculum concentration resulted in a smaller amount of inactivation by citral. According to these authors, for high cell concentrations up to 10^8^ and 10^9^ cfu/ml, more citral would be needed to kill the same proportion of *E. coli* cells. These observations are important because predictive growth model assays are typically performed using initial bacterial concentrations higher than 10^3^ cfu/mL. Our results demonstrate the importance of minimising the initial contamination level of the raw material to ensure the effectiveness of citral as an antimicrobial agent by increasing the lag phase.

The effect of the recovery medium on the behaviour of both species was also studied by recovering the untreated and stressed cells on nonselective or “reference” medium, which enumerates the entire population, and on selective medium, which only recovers the undamaged cells.

No significant differences were observed in the duration of the lag phase (λ) and the growth rate (μ_max_) of untreated *Listeria innocua* and *Listeria monocytogenes* cells as a function of the recovering medium (i.e., nonselective TSA-YE and selective TSA-YE-SC) at either inoculum concentrations (p > 0.05), as presented in Tables 3 and 4. Therefore, the salt concentration that was used as a supplement in the culture medium had no effect on the growth kinetics of untreated cells at either inoculum size.

**Table 3:** Mean values ± standard error of the duration of the lag phase (λ) and the maximum specific growth rate (μ_max_) of *Listeria monocytogenes* cells as a function of the recovery culture medium (i.e., TSA-YE and TSA-YE-SC) and the concentration of citral using an initial inoculum size of 10^2^ cfu/mL and of 10^6^ cfu/mL.

**Citral (μL/mL)**	10^2^ cfu/mL			
	**λ ± SE (h)**		**μ_max_ ± SE (log_10_ (cfu/mL))/h**	
	**TSA-YE-SC**	**TSA-YE**	**TSA-YE-SC**	**TSA-YE**
**0.0**	0.520 ± 0.02^A^ ^a^	0.533± 0.03^A^ ^a^	0.601 ± 0.05^A^ ^a^	0.590 ± 0.04^A^ ^a^
**0.150**	1.802 ± 0.05^B^ ^b^	2.569 ± 0.04^B^ ^a^	0.525 ± 0.02^B^ ^b^	0.470 ± 0.01^B^ ^a^
**0.250**	7.010 ± 0.08^C^ ^b^	8.094 ± 0.04^C^ ^a^	0.216 ± 0.05^C^ ^b^	0.181 ± 0.01^C^ ^a^
	10^6^ cfu/mL			
	**λ ± SE (h)**		**μ_max_ ± SE (log_10_ (cfu/mL))/h**	
	**TSA-YE-SC**	**TSA-YE**	**TSA-YE-SC**	**TSA-YE**
**0.0**	0.490 ± 0.06^A^ ^a^	0.500 ± 0.04^A^ ^a^	0.582 ± 0.08^A^ ^a^	0.571 ± 0.03^A^ ^a^
**0.150**	0.967 ± 0.04^B^ ^b^	1.259 ± 0.06^B^ ^a^	0.498 ± 0.01^B^ ^b^	0.448 ± 0.01^B^ ^a^
**0.250**	3.987 ± 0.03^C^ ^b^	4.754 ± 0.05^C^ ^a^	0.142 ± 0.06^C^ ^a^	0.102 ± 0.02^C^ ^a^

^A–C^Mean values followed by different letters in the same column differ significantly by Fisher’s LSD test (p ≤ 0.05).

^a, b^Mean values followed by different letters in the same row differ significantly by Fisher’s LSD test (p ≤ 0.05).

**Table 4:** Mean values ± standard error of the duration of the lag phase (λ) and the maximum specific growth rate (μ_max_) of *Listeria innocua* as a function of the recovery culture medium (i.e., TSA-YE and TSA-YE-SC) and the concentration of citral using an initial inoculum size of 10^2^ cfu/mL and of 10^6^ cfu/mL.

**Citral (μL/mL)**	10^2^ cfu/mL			
	**λ ± SE (h)**		**μ_max_ ± SE (log_10_ (cfu/mL))/h**	
	**TSA-YE-SC**	**TSA-YE**	**TSA-YE-SC**	**TSA-YE**
**0.0**	0.300 ± 0.04^A^ ^a^	0.305 ± 0.03^A^ ^a^	0.569 ± 0.02^A^ ^a^	0.547 ± 0.02^A^ ^a^
**0.150**	1.727 ± 0.01^B^ ^b^	2.180 ± 0.01^B^ ^a^	0.524 ± 0.01^B^ ^b^	0.440 ± 0.01^B^ ^a^
**0.250**	5.315 ± 0.01^C^ ^b^	6.012 ± 0.01^C^ ^a^	0.309 ± 0.02^C^ ^b^	0.202 ± 0.04^C^ ^a^
	10^6^ cfu/mL			
	**λ ± SE (h)**		**μ_max_ ± SE (log_10_ (cfu/mL))/h**	
	**TSA-YE-SC**	**TSA-YE**	**TSA-YE-SC**	**TSA-YE**
**0.0**	0.276 ± 0.06^A^ ^a^	0.287 ± 0.07^A^ ^a^	0.533 ± 0.03^A^ ^a^	0.529 ± 0.04^A^ ^a^
**0.150**	0.855 ± 0.01^B^ ^b^	1.832 ± 0.01^Ba^	0.488 ± 0.01^B^ ^b^	0.432 ± 0.01^Ba^
**0.250**	3.889 ± 0.02^C^ ^b^	4.100 ± 0.03^C^ ^a^	0.123 ± 0.03^C^ ^a^	0.101 ± 0.03^C^ ^a^

^A–C^Mean values followed by different letters in the same column differ significantly by Fisher’s LSD test (p ≤ 0.05).

^a, b^Mean values followed by different letters in the same row differ significantly by Fisher’s LSD test (p ≤ 0.05).

When *Listeria innocua* and *Listeria monocytogenes* cells were stressed by different citral concentrations, the results indicated that for both initial inoculum concentrations, *L. innocua* and *L. monocytogenes* cells that were recovered in TSA-YE exhibited a greater extension of the lag time and a slower growth rate than those recovered in TSA-YE-SC (p ≤ 0.05). However, when the higher inoculum size was grown with the highest concentration of citral, no significant differences in the maximum specific growth rate were observed (Tables 3 and 4). The extension of the lag phase in cells recovered in reference medium could be due to the growth of a mixture of healthy and damaged cells, while in the stressing medium, only healthy cells can grow normally, as demonstrated in assays using unstressed cells. The extension of the lag phase was also observed by [41], [42] when the cells were physically injured. [43] also reported that sublethally injured *L. monocytogenes* often exhibit a slower growth rate than their healthy counterparts, as well as altered virulence characteristics and higher sensitivity to unfavourable conditions.

Considering the results presented in Tables 3 and 4, the percentage of damaged cells was also determined. The results indicated that the presence of citral in the TSB culture medium damaged cells; this damage was dependent on both citral concentration and inoculum concentration (Table 5). For both *Listeria* species, the higher inoculum concentration resulted in a lower percentage of damaged cells, except for untreated cells, for which no significant differences were obtained (p>0.05). When bacterial cells were exposed to citral, the proportion of sublethally injured cells increased with increasing citral doses; the one-way ANOVA revealed significant differences between citral levels (p ≤ 0.05) for both *Listeria* species and both inoculum concentrations. Our results are consistent with [40], who detected increased proportions of sublethally damaged *E. coli* in the presence of 200 μL/L of citral.

**Table 5:** Exposure of *Listeria innocua* and *Listeria monocytogenes* to citral during 30 h on percentage damage recovered in a complete medium (TSA-YE) with different inoculum sizes (10^2^ cfu/mL and 10^6^ cfu/mL) at time 0.

**Citral (μL/mL)**	**Percentage of sublethally injured cell of *Listeria innocua***		**Percentage of sublethally injured cell of *Listeria monocytogenes***	
	**10^2^ Inoculum concentration**	**10^6^ Inoculum concentration**	**10^2^ Inoculum concentration**	**10^6^ Inoculum concentration**
**0.0**	0.510^A^ ^a^	0.493^A^ ^a^	0.492^A^ ^a^	0.481^A^ ^a^
**0.150**	2.407^B^ ^a^	0.775^Bb^	2.465^B^ ^a^	0.823^Bb^
**0.250**	3.612^C^ ^a^	2.370^C^ ^b^	6.257^C^ ^a^	2.626^C^ ^b^

^A–C^Mean values followed by different letters in the same column differ significantly by Fisher’s LSD test (p ≤ 0.05).

^a, b^Mean values followed by different letters in the same row differ significantly by Fisher’s LSD test (p ≤ 0.05).

The damage cell membrane of *Listeria* by citral can also be supported by other experimental methods. The overview of experimental approaches used to identify target sites and modes of action of antimicrobial compounds includes monitoring of disruption of cytoplasmic membrane by uptake of fluorescent DNA-binding stains, such as propidium iodide (PI), SYTO9, ethidium bromide (EB), and carboxy fluorescein diacetate (cFDA), using fluorescence microscopy or flow cytometry [44], by measurement of ATP leakage from the cells using an assay based on luciferase activity quantified bybioluminescence [10], [45], by changes in concentration gradientes of ions across a cell membrane, which can be detected by fluorometry using bis-oxonol or DiSC3, or by flow cytometry using bis-oxonol, DiOC2, or BOX [10] and others methods.

## Conclusions

The results presented here demonstrate that citral exhibited antilisterial activity against *L. innocua* and *L. monocytogenes* and can be applied in active packaging to control possible recontamination of foods or in combination with other preservation technologies. It is necessary to take into account that its application as food preservative alone is limited by its strong flavor when added in large amounts, which negatively affects the organoleptic properties of food. So, it is necessary to combine these main components of EOs to decrease their addition to produce the desired antibacterial effect at a concentration that does not produce undesirable changes in the flavor or aroma. Nevertheless, low concentrations of citral as those used when combined with other preservation technologies produce damaged cells. This result is important because damaged cells can contribute to the creation of resistance or changes in the virulence of the cells. The effect of citral on microorganisms was dependent on the concentration of preservative present in the culture medium and on the inoculum concentration being this an important parameter to be considered during the development of growth models. *Listeria innocua* could be used as surrogate for *Listeria monocytogenes* when testing the effect of this antimicrobial because *Listeria innocua* represents a worse scenario due to its shorter lag phase.

## References

[1] European Commission (EC) (2005) Commission Regulation (EC) No 2073/2005 of 15 November (2005) on microbiological criteria for foodstuffs. Brussels: European Commission. Available online: http://eur-lex.europa.eu/LexUriServ/LexUriServ.do?uri=CELEX:32005R2073:EN:HTML. Last accessed at 24. October 2014.

[2] Ryser E, Marth E (Eds.) (1999). *Listeria*, Listeriosis, and Food Safety, 2nd Ed. New York: Marcel Dekker.

[3] Fairchild TM, Foegeding PM (1993) A proposed nonpathogenic biological indicator for thermal inactivation of *Listeria monocytogenes* . Appl Environ Microbiol 59, 1247–1250. 8489233 10.1128/aem.59.4.1247-1250.1993 PMC202269

[4] Barba FJ, Criado MN, Belda-Galbis CM, Esteve MJ, Rodrigo D (2014) Stevia rebaudiana Bertoni as a natural antioxidant/antimicrobial for high pressure processed fruit extract: Processing parameter optimization. Food Chem 148, 261–267. 10.1016/j.foodchem.2013.10.048 24262555

[5] Pina-Pérez MC, Silva-Angulo AB, Rodrigo D, Martinez A (2009) Synergistic effect of pulsed electric fields and cocoanOX 12% on the inactivation kinetics of *Bacillus cereus* in a mixed beverage of liquid whole egg and skim milk. Int J Food Microbiol 139, 196–204. 10.1016/j.ijfoodmicro.2009.01.021 19232768

[6] Hyldgaard M, Mygind T, Meyer RL (2012) Essential oils in food preservation: mode of action, synergies, and interactions with food matrix componentes. Front Microbiol 3, 1–24. 10.3389/fmicb.2012.00012 22291693 PMC3265747

[7] Benvenuti F, Gironi F, Lamberti LX (2001) Supercritical deterpenation of lemon essential oil, experimental data and simulation of the semicontinuous extraction process. J Supercrit Fluids 20: 29–44. 10.1016/S0896-8446(01)00058-4

[8] Fisher K, Phillips C (2006) The effect of lemon, orange and bergamot essential oils and their components on the survival of *Campylobacter jejuni*, *Escherichia coli* O157, *Listeria monocytogenes*, *Bacillus cereus* and *Staphylococcus aureus in vitro* and in food systems. J Appl Microbiol 101, 1232–1240. 10.1111/j.1365-2672.2006.03035.x 17105553

[9] Fisher K, Phillips C (2008) Potential antimicrobial uses of essential oils in food: is citrus the answer? Trends Food Science Technol 19, 156–164. 10.1016/j.tifs.2007.11.006

[10] Fisher K, Phillips C (2009) The mechanism of action of a citrus oil blend against *Enterococcus faecium* and *Enterococcus faecalis* . J Appl Microbiol 106, 1343–1349. 10.1111/j.1365-2672.2008.04102.x 19187138

[11] Walsh SE, Maillard JY, Russell AD, Charbonneau D, Bartolo RG, et al (2003) Development of bacterial resistance to several biocides and effects on antibiotic susceptibility. J Hosp Infect 55, 98–107. 10.1016/S0195-6701(03)00240-8 14529633

[12] Lado B, Yousef A 2002 Alternative food preservation technologies: efficacy and mechanisms. Microbes Infect 4, 433–440. 10.1016/S1286-4579(02)01557-5 11932194

[13] Yousef A, Juneja VK (2003) Microbial stress adaptation and food safety. CRC Press, Boca Raton, FL, USA.

[14] Augustin JC, Brouillaud-Delattre A, Rosso L, Carlier V (2000) Significance of inoculum size in the lag time of *Listeria monocytogenes* . Appl Environ Microbiol. 66, 1706–1710. 10.1128/AEM.66.4.1706-1710.2000 10742265 PMC92046

[15] Robinson TP, Aboaba OO, Kaloti A, Ocio MJ, Baranyi J, et al (2001) The effect of inoculum size on the lag phase of *Listeria monocytogenes* . Int J Food Microbiol 70, 163– 173. 10.1016/S0168-1605(01)00541-4 11759754

[16] Saucedo-Reyes D, Marco-Celdrán A, Pina-Pérez MC, Rodrigo D, Martínez López A (2009) Modeling survival of high hydrostatic pressure treated stationary and exponential phase *Listeria innocua* cells. Innov Food Sci Emerg Technol 10, 135–141. 10.1016/j.ifset.2008.11.004

[17] Belda-Galbis CM, Martínez A, Rodrigo D (2011) Effect of carvacrol on *Listeria innocua* growth at different incubation temperatures in reference media. Paper presented at: 1st CIGR International Workshop on Food Safety: Advances and Trends; 14th-15th 4; Dijon (France).

[18] Belda-Galbis CM, Martínez A, Rodrigo D (2011) In: UV, UPV (Eds.) Proceedings of the VI Congreso Nacional de Ciencia y Tecnología de los Alimentos. Valencia (Spain): Universitat Politècnica de València Chapter: Seguridad, Evaluación in vitro de la actividad antimicrobiana del citral sobre Listeria innocua a distintas temperaturas.

[19] Silva-Angulo A, Belda-Galbis CM, Zanini SF, Rodrigo D, Martorell P, et al (2012) Sublethal Damage in: *Listeria monocytogenes* after non-thermal treatments, and implications for food safety. In: Romano A.; Giordano C.F. (Eds.) *Listeria* infections: Epidemiology, pathogenesis and treatment. (pp. 99–114), Nova Science Publishers Inc.

[20] Gibson AM, Bratchell N, Roberts TA (1988) Predicting microbial growth: Growth responses of salmonellae in a laboratory medium as affected by pH, sodium chloride and storage temperature. Int J Food Microbiol 6, 155–178. 10.1016/0168-1605(88)90051-7 3275296

[21] Ferrer C, Ramón D, Mugüerza B, Marco A, Martinez A (2009) Effect of olive powder on the growth inhibition of *Bacillus cereus* . Foodborne Pathog Dis 6, 33–37. 10.1089/fpd.2008.0133 19061367

[22] Guillier L, Pardon P, Augustin J C (2005) Influence of stress on individual lag time distributions of *Listeria monocytogenes* . Appl Environ Microbiol 71, 2940–2948. 10.1128/AEM.71.6.2940-2948.2005 15932988 PMC1151854

[23] McMeekin TA, Olley JN, Ross T, Ratkowsky DA (1993) Predictive Microbiology - Theory and Application. Somerset, UK: Research Studies Press Ltd.

[24] Arroyo C, Somolinos M, Cebrian G, Condon S, Pagan R (2010) Pulsed electric fields cause sublethal injuries in the outer membrane of *Enterobacter sakazakii* facilitating the antimicrobial activity of citral. Lett Appl Microbiol 51, 525–531. 10.1111/j.1472-765X.2010.02931.x 21039664

[25] Mackey BM (2000) Injured bacteria. In: Lund A. M.; Baird-Parker T. C.; Gould G. W. (Eds.) The microbiological safety and quality of food. v. 1. Cap. 15, (pp. 315–354). New York: Springer.

[26] Busch SV, Donnelly CW (1992) Development of a repair-enrichment broth for resuscitation of heat-injured *Listeria monocytogenes* and *Listeria innocua* . Appl Environ Microbiol 58, 14–20. 1531746 10.1128/aem.58.1.14-20.1992 PMC195165

[27] Dykes GA, Withers KM (1999) Sub-lethal damage of *Listeria monocytogenes* after long-term chilled storage at 4°C. Lett Appl Microbiol 28, 45–48. 10.1046/j.1365-2672.1999.00472.x 10030031

[28] Klein G, Rüben C, Upmann M (2013) Antimicrobial activity of essential oil components against potential food spoilage microorganisms. Curr Microbiol 67, 200–208. 10.1007/s00284-013-0354-1 23503789

[29] Si W, Gong J, Tsao R, Zhou T, Yu H, et al (2006) Antimicrobial activity of essential oils and structurally related synthetic food additives towards selected pathogenic and beneficial gut bacteria. J Appl Microbiol 100, 296–305. 10.1111/j.1365-2672.2005.02789.x 16430506

[30] Augustin JC, Carlier V (2000) Mathematical modelling of the growth rate and lag time for *Listeria monocytogenes* . Int J Food Microbiol 56, 29–51. 10.1016/S0168-1605(00)00223-3 10857924

[31] Bloomfield SF (1991) Mechanisms of action of chemical biocides. Their study and exploitation. In: Denyer S.P., Hugo W.B. (Eds). Methods for assessing antimicrobial activity. Technical series of the Society for Applied Bacteriology, Oxford, UK Blackwell Scientific Publications.

[32] Encinas JP, Sanz JJ, Garcia-Lopez ML, Otero A (1999) Behaviour of *Listeria* spp. in naturally contaminated chorizo (Spanish fermented sausage). Int J Food Microbiol 46, 167–171. 10.1016/S0168-1605(98)00184-6 10728617

[33] Berends BR, vanKnapen F, Snijders JMA, Mossel DAA (1997) Identification and quantification of risk factors regarding *Salmonella* spp. on pork carcasses. Int J Food Microbiol 36, 199–206. 10.1016/S0168-1605(97)01267-1 9217109

[34] FAO/OMS (2004) Evaluación de riesgos de *Listeria monocytogenes* en alimentos listos para el consumo—Resumen interpretativo. Serie sobre Evaluación de riesgos microbiológicos. n. 4. 54p.

[35] McLauchlin J (1996) The relationship between *Listeria* and listeriosis. Food Control 7, 187–193.

[36] FDA/FSIS (2003) Quantitative assessment of the relative risk to public health from foodborne *Listeria monocytogenes* among selected categories of read-to-eat foods. Available online: http://www.fda.gov/downloads/food/scienceresearch/researchareas/riskassessmentsafetyassessment/ucm197330.pdf. Last accessed at 24. October 2014.

[37] Baranyi J (1998) Comparison of stochastic and deterministic concepts of bacterial lag. J Theor Biol 192, 403–408. 10.1006/jtbi.1998.0673 9735256

[38] Stephens PJ, Joynson JA, Davies KW, Holbrook R, Lappin-Scott HM, et al (1997) The use of an automated growth analyser to measure recovery times of single heat-injured *Salmonella* cells. J Appl Microbiol 83, 445–455. 10.1046/j.1365-2672.1997.00255.x 9351226

[39] Coleman ME, Tamplin ML, Phillips JG, Marmer BS (2003) Influence of agitation, inoculum density, pH, and strain on the growth parameters of *Escherichia coli* O157:H7 relevance to risk assessment. Int J Food Microbiol 83, 147–160. 10.1016/S0168-1605(02)00367-7 12706036

[40] Somolinos M, Garcıa D, Condon S, Mackey B, Pagan R (2010) Inactivation of *Escherichia coli* by citral. J Appl Microbiol 108, 1928–1939. 19891710 10.1111/j.1365-2672.2009.04597.x

[41] Mackey BM, Derrick CM (1982) The effect of sublethal injury by heating, freezing, drying and gamma-radiation on the duration of the lag phase of *Salmonella typhimurium* . J Appl Bacteriol 53, 243–251. 10.1111/j.1365-2672.1982.tb04683.x 6761334

[42] Mackey BM, Derrick CM (1984) Conductance measurements of the lag phase of injured *Salmonella typhimurium* . J Appl Bacteriol 57, 299–308. 10.1111/j.1365-2672.1984.tb01394.x 6389465

[43] Buncic S, Avery SM (1996) Relationship between variations in pathogenicity and lag phase at 37°C of *Listeria monocytogenes* previously stored at 4°C. Lett Appl Microbiol 23, 18–22. 10.1111/j.1472-765X.1996.tb00020.x 8679139

[44] Gill AO, Holley RA (2006) Disruption of *Escherichia coli*, *Listeria monocytogenes* and *Lactobacillus sakei* cellular membranes by plant oil aromatics. Int J Food Microbiol 108, 1–9. 10.1016/j.ijfoodmicro.2005.10.009 16417936

[45] Gill AO, Holley RA (2006) Inhibition of membrane bound ATP ases of *Escherichia coli* and *Listeria monocytogenes* by plant oil aromatics. Int J Food Microbiol 111, 170–174. 10.1016/j.ijfoodmicro.2006.04.046 16828188

